# Effects of 16S rDNA sampling on estimates of the number of endosymbiont lineages in sucking lice

**DOI:** 10.7717/peerj.2187

**Published:** 2016-07-19

**Authors:** Julie M. Allen, J. Gordon Burleigh, Jessica E. Light, David L. Reed

**Affiliations:** 1 Illinois Natural History Survey, University of Illinois at Urbana-Champaign, Champaign, IL, United States; 2 Florida Museum of Natural History, University of Florida, Gainesville, FL, United States; 3 Department of Biology, University of Florida, Gainesville, FL, United States; 4 Department of Wildlife and Fisheries Sciences, Texas A&M University, College Station, TX, United States

**Keywords:** Phylogenetics, 16S rDNA, *Gammaproteobacteria*, Endosymbiosis, Anoplura, Sucking lice, Endosymbiont

## Abstract

Phylogenetic trees can reveal the origins of endosymbiotic lineages of bacteria and detect patterns of co-evolution with their hosts. Although taxon sampling can greatly affect phylogenetic and co-evolutionary inference, most hypotheses of endosymbiont relationships are based on few available bacterial sequences. Here we examined how different sampling strategies of *Gammaproteobacteria* sequences affect estimates of the number of endosymbiont lineages in parasitic sucking lice (Insecta: Phthirapatera: Anoplura). We estimated the number of louse endosymbiont lineages using both newly obtained and previously sequenced 16S rDNA bacterial sequences and more than 42,000 16S rDNA sequences from other *Gammaproteobacteria*. We also performed parametric and nonparametric bootstrapping experiments to examine the effects of phylogenetic error and uncertainty on these estimates. Sampling of 16S rDNA sequences affects the estimates of endosymbiont diversity in sucking lice until we reach a threshold of genetic diversity, the size of which depends on the sampling strategy. Sampling by maximizing the diversity of 16S rDNA sequences is more efficient than randomly sampling available 16S rDNA sequences. Although simulation results validate estimates of multiple endosymbiont lineages in sucking lice, the bootstrap results suggest that the precise number of endosymbiont origins is still uncertain.

## Introduction

There are many types of associations of bacteria with insects, including obligate mutualistic endosymbiotic bacteria, which inhabit specialized cells and provide a variety of benefits to their insect hosts (Buchner, 1965; Moran, McCutcheon & Nakabachi, 2008), as well as pathogenic bacteria, which can cause not only disease but also morphological (e.g., Werren, Baldo & Clark, 2008) and behavioral changes in their host (e.g., Zchori-Fein et al., 2001). Phylogenetic trees have revealed intricate and complicated co-evolutionary histories of insects and their associated bacteria (Buchner, 1965; Moran, McCutcheon & Nakabachi, 2008). For example, the obligate mutualistic bacteria that reside in specialized host cells often have matching topologies with their hosts, suggesting they are vertically transmitted and have had a long-term co-evolutionary history (Moran & Baumann, 1994). Although phylogenetic trees are crucial for understanding these evolutionary patterns, the effects of taxonomic sampling, especially in regard to the hyper-diverse and yet largely unknown bacteria, on co-evolutionary inference has not been examined extensively. Because our interpretations of the co-evolutionary history of the bacteria and their host depend on these trees, it is imperative that bacterial phylogenetic trees are built using appropriate sampling and methodologies.

Arguably, bacteria are one of the more challenging groups for phylogenetic inference. Although there currently are ∼25 million eubacteria sequences in NCBI, the majority of the data is from 16S rDNA. In fact, most of our understanding of the diversity and phylogeny of bacteria is based solely on this locus (Schloss & Handelsman, 2004; Lozupone & Knight, 2007). For example, most environmental studies identify bacterial diversity by sequencing a small section of the 16S rDNA gene and comparing it to the enormous number of 16S rDNA sequences that reside in public databases (Hamaday, Louzupone & Knight, 2010; Li & Godzik, 2006). In the case of endosymbiotic bacteria, the majority of studies use only a handful of representative bacterial sequences to build phylogenetic trees despite the vast array of sequences available, possibly due to the difficulties of large alignments and building large phylogenetic trees.

While taxon sampling can greatly affect phylogenetic estimates (e.g., Hills, 1996; Hillis, 1998; Pollock et al., 2002; Zwickl & Hillis, 2002; Heath, Hedtke & Hillis, 2008), few studies of insect endosymbionts have examined how taxon sampling may affect the phylogenetic hypothesis, particularly with the massive number of 16S rDNA sequences available for sampling. Assuming that endosymbiosis is non-reversible trait (i.e., endosymbionts can’t revert back to non-endosymbionts), then the number of endosymbiont origins is simply the number of independent endosymbiont clades in a bacterial phylogeny. Sampling both endosymbiont and non-endosymbiont lineages in a phylogenetic analysis can affect estimates of the number of endosymbiont origins either by the insertion of new sequences, which can break up or create clades of endosymbionts, or by directly affecting the topology of the phylogenetic tree. In this study, we sample from the 16S rDNA database to determine how taxon sampling affects estimates of the number of distinct endosymbiotic bacterial lineages found within parasitic sucking lice (Phthiraptera: Anoplura) and our interpretations of the coevolutionary history between these two lineages.

### Study system

Sucking lice are wingless, blood-feeding insects that parasitize eutherian mammals. These lice have endosymbiotic bacteria that synthesize necessary amino acids and vitamins absent from the louse’s diet and are therefore thought to be required for louse survival (Buchner, 1965; Puchta, 1955). Previous studies have indicated that there are at least six different lineages (i.e., independent origins) of endosymbionts in sucking lice, all of which reside within *Gammaproteobacteria*, a class of gram-negative bacteria (Sasaki-Fukatsu et al., 2006; Allen et al., 2007; Hypsa & Krízek, 2007; Allen et al., 2009; Fukatsu et al., 2009; Perotti et al., 2009). Phylogenetic studies show little concordance between the louse and bacteria trees (Hypsa & Krízek, 2007; Allen et al., 2009); however, these studies estimated the number of louse endosymbiont lineages from only a tiny fraction (e.g., ∼33–46 sequences) of the available 16S rDNA sequences.

Here, we assembled a dataset with both new and previously studied 16S rDNA sequence data from sucking louse endosymbionts and ∼42,000 publicly available *Gammaproteobacteria* 16S rDNA sequences to determine the effect of sampling on our estimates of endosymbiont diversity. We estimated the number of independent endosymbiont lineages on phylogenetic trees constructed from subsets of the entire sample of sequences. These subsets were created by either randomly sampling sequences or sampling sequences by maximizing genetic diversity. Lastly, we performed both parametric and nonparametric bootstrapping approaches to examine uncertainty and possible error in these estimates.

## Materials and Methods

### Louse endosymbiont sampling and sequencing

We obtained 23 louse specimens, representing 8 families and 21 species, from museums and mammal collectors (Table 1). The majority of these samples were obtained via requests for donations from the mammal community; therefore, our sampling is limited to donations we received. The lice were washed three times in 500 ul of 5% bleach and two times with sterile water to remove external bacteria (e.g., Meyer & Hoy, 2008). Lice were crushed and DNA extracted using a Qiagen micro kit (Cat No. 56304). We followed the manufacturer’s protocol, except that the lice were placed in 80 ul of Proteinase K (Qiagen) and incubated overnight on a heating block at 55°C, and the DNA was eluted in 50 ul of sterile water. Water was used as a negative control for every extraction to ensure that there was no bacterial contamination. We amplified 16S rDNA from putative bacterial endosymbionts from each of the DNA samples using Stratagene Hi-Fidelity Master Mix (Cat No. 600650-51) with general bacterial primers 27F and either 1525R or 1329R (Lane, 1991) at a final concentration of 0.7 uM and total reaction volume of 50 ul. Polymerase chain reaction (PCR) cycling conditions included an initial denaturation step at 95°C for two minutes followed by 40 cycles of denaturation at 95°C for 40 s, annealing at 50°C for 30 s, and extension at 72°C for two minutes, and a final extension step at 72°C for 30 min. The resulting PCR products were cloned using the Invitrogen Cloning Kit (Cat No. 45-0030), and 96 colonies per specimen were picked and sequenced at the University of Florida ICBR sequencing facility. The resulting 16S rDNA sequences were ∼1,300 base pairs in length. All sequences have been submitted to GenBank (Accession numbers: KX146199–KX146216).

**Table 1:** Table of Anoplura endosymbiont sequences. Family and species of sucking lice (Phthiraptera: Anoplura) from which endosymbionts were targeted. Also indicated are the collection locality, louse taxon label (for use in the laboratory), mammalian host, presence of putative endosymbiont (where the superscript “b” indicates that *Bartonella*, a louse pathogen, was sequenced), percent AT content, if the top hit from a BLAST search was an endosymbiont, and finally, if the top hit from the BLAST search was an endosymbiont from a sucking louse.

Louse family and species Country and State^a^	Taxon label	Host (Order: Family) Museum Voucher (if known)^b^	Endosymbiont present	%AT	BLAST endosymbiont	BLAST Anoplura
**Echinophthiriidae**						
*Proechinophthirus fluctus* (USA: AK)	Echin3.17.09.2	*Callorhinus ursinus* (Carnivora: Otariidae)	Yes	45%	Yes	No
**Haematopinidae**						
*Haematopinus suis* (USA: FL)	Hpsu7.14.09.4	*Sus scrofa* (Artiodactyla: Suidae)	Yes	52%	Yes	Yes
**Hoplopleuridae**						
*Ancistroplax crocidurae* 1 (Vietnam)	Axcro4.26.09.1	*Crocidura* sp. (Soricomorpha: Soricidae)	Yes	50%	Yes	No
*Ancistroplax crocidurae* 2 (China)	Axsp7.14.09.5	*Crocidura attenuata* (Soricomorpha: Soricidae)	Yes (2)	49%, 45%	Yes, Yes	No, No
*Hoplopleura ferrisi* 2 (MX: Puebla)	Hofer7.14.09.8	*Peromyscus difficilis* (Rodentia: Cricetidae; LSUMZ 36247)	No	–		
*Hoplopleura hirsuta* (USA: TX)	Hosp4.17.09.7	*Sigmodon hispidus* (Rodentia: Cricetidae; LSUMZ 36377)	No	–		
*Hoplopleura onychomydis* (USA: AZ)	Hoony8.27.08.6	*Onychomys torridus* (Rodentia: Cricetidae; NMMNH 4394)	No	–		
*Hoplopleura reithrodontomydis* 2 (USA: AZ)	Hosp7.14.09.6	*Reithrodontomys* sp. (Rodentia: Cricetidae; NMMNH 4411)	No	–		
*Hoplopleura sicata* (China)	Hosic7.14.09.9	*Niviventer fulvescens* (Rodentia: Muridae)	No	–		
**Linognathidae**						
*Linognathus spicatus* (Zimbabwe)	Linog6.22.09.1	*Connochaetes taurinus* (Artiodactyla: Bovidae)	Yes	52%	Yes	No
**Pedicinidae**						
*Pedicinus pictus* 1 (Ivory Coast)	Qnpic3.31.08.1	*Piliocolobus badius* (Primates: Cercopithecidae)	Yes	54%	Yes	Yes
*Pedicinus pictus* 2 (Ivory Coast)	Qnpic6.30.09.2	*Colobus polykomos* (Primates: Cercopithecidae)	Yes	53%	Yes	Yes
*Pedicinus pictus* 3 (Ivory Coast)	Qnsp3.31.08.3	*Colobus polykomos* (Primates: Cercopithecidae)	Yes	54%	Yes	Yes
**Pediculidae**						
*Pediculus humanus capitis* (USA: FL)	Pdcap9.20.05.2NW	*Homo sapiens* (Primates: Hominidae)	Yes	51%	Yes	Yes
*Pediculus humanus humanus* (USA: MD)	Pdhum5.19.05.2	*Homo sapiens* (Primates: Hominidae)	Yes	51%	Yes	Yes
**Polyplacidae**						
*Fahrenholzia ehrlichi* 1 (USA: TX)	Fzehr8.20.08.1	*Liomys irroratus* (Rodentia: Heteromyidae; LSUMZ 36395)	Yes	52%	Yes	No
*Fahrenholzia ehrlichi* 2 (MX:Puebla)	Fzehr6.30.09.4	*Liomys irroratus* (Rodentia: Heteromyidae; LSUMZ 36299)	Yes	51%	Yes	No
*Linognathoides marmotae* 1 (USA: CO)	Lnlae6.30.09.3	*Marmota flaviventris* (Rodentia: Sciuridae)	Yes	54%	Yes	No
*Lemurpediculus verruculosus* 1 (Madagascar)	Lesp4.26.09.2	*Microcebus rufus* (Primates: Cheirogaleidae)	Yes	53%	Yes	No
*Neohaematopinus sciuropteri* (USA: OR)	Nescp6.30.09.5	*Glaucomys sabrinus* (Rodentia: Sciuridae)	Yes	53%	Yes	No
*Neohaematopinus neotomae* (USA: CA)	Neneo8.20.08.2	*Neotoma lepida* (Rodentia: Cricetidae; MLZ 1921)	No	–		No
*Sathrax durus* (Vietnam)	Sathrax4.26.09.3	*Tupaia belangeri* (Scandetia: Tupaiidae)	Yes	45%	Yes	No
**Pthiridae**						
*Pthirus gorillae* (Uganda)	Ptgor9.14.08.1	*Gorilla gorilla* (Primates: Hominidae)	Yes	53%	Yes	Yes

**Notes.**

a USA, United States (AK, Alaska; AZ, Arizona; CA, California; CO, Colorado; FL, Florida; MD, Maryland; OR, Oregon; TX, Texas); MX, Mexico.

b MLZ, Moore Laboratory of Zoology; LSUMNZ, Louisiana State University Museum of Natural Science; NMMNH, New Mexico Museum of Natural History.

We assessed if the 16S rDNA sequences amplified by PCR from the louse specimens came from endosymbionts based on their similarity to other endosymbiont sequences. If the most similar sequence from a BLAST search (Altschul et al., 1990) of the non-redundant nucleotide database in GenBank was from an endosymbiont, we identified the sequences as endosymbionts.

We also downloaded from GenBank 12 endosymbiont sequences from sucking lice and Rhynchophthirina chewing lice (Accessions: DQ076661, DQ076662, DQ076665, DQ076664, EU827263, AB478979, EF110571, EF110573, DQ076667, DQ076666, EF110571, DQ076663; Hypsa & Krízek, 2007; Allen et al., 2009; Fukatsu et al., 2009). Rhynchophthirina is a suborder of blood-feeding chewing lice that is the sister group to Anoplura (Cruickshank et al., 2001; Barker et al., 2003; Johnson, Yoshizawa & Smith, 2004; Yoshizawa & Johnson, 2010). Members of the suborder Rhynchophthirina parasitize eutherian mammals and are thought to have an endosymbiont serving a similar function as those in sucking lice (Ries, 1931).

### 16S rDNA sampling and alignments

We obtained an initial alignment of ∼72,000 *Gammaproteobacteria* 16S rDNA sequences from the Ribosomal Database Project (Cole et al., 2005; http://rdp.cme.msu.edu/). We removed any sequences that contained fewer than 750 nucleotides and then deleted any columns in the alignment that contained fewer than 100 nucleotides. Next, we removed extra copies of identical sequences, so each remaining sequence was unique. We first aligned the entire dataset along with the endosymbiont sequences with MUSLCE (Edgar, 2004), but we found obvious anomalies in the resulting alignment (e.g., stem regions were not adequately aligned, likely due to difficulties aligning the more variable loop regions across such a large alignment). To ameliorate errors in the automated alignment, the sequences were first split into 20 clusters of approximately equal size (ca. 2,000 sequences), and each cluster was aligned using the default settings in MUSCLE. The resulting alignments were manually checked by multiple individuals (see ‘Acknowledgements’) and verified by JMA. Profile alignments were then created using MUSCLE to combine the edited alignments. The resulting alignment of all sequences was again checked by eye by JMA. Regions of ambiguous alignment were removed, and any extra identical sequences were pruned from the alignment. This resulted in a final alignment of 42,626 sequences that was 1,476 characters in length. The final alignment is available in the Dryad repository (Dryad DOI: 10.5061/dryad.db0r1).

To determine how taxon sampling affects estimates of the number of endosymbiont lineages, we assembled five subsets of the 16S rDNA alignment. Our goal was to create taxonomic subsamples of increasing size such that each reflected the breadth of genetic diversity in the full alignment. To do this, we first clustered the sequences based on similarity using the QT-clustering algorithm (Heyer, 1999) implemented in RAxML-VI-HPC version 7.0.4 (Stamatakis, 2006). We used five different thresholds for the sequence similarity clustering: 70%, 80%, 85%, 90%, and 95%. A higher threshold results in more, smaller clusters composed of more similar sequences. For each threshold, we sampled at least one sequence per cluster while ensuring that each subsample contained all louse endosymbiont sequences and all sequences included in the smaller clusters (e.g., the 85% cluster contained all sequences in the 80% cluster, the 80% cluster contained all sequences in the 70% cluster, etc.). In total, the sizes of the subsampled datasets were 39 taxa (70% cluster), 76 taxa (80% cluster), 217 taxa (85% cluster), 865 taxa (90% cluster) and 4,275 taxa (95% cluster). In order to compare this sampling strategy to a random taxon sampling strategy, we also created 100 datasets each of 76, 217, 865, and 4,275 taxa, each including all louse endosymbiont sequences with the remaining sequences randomly selected from the full alignment.

### Phylogenetic analysis

We performed maximum likelihood (ML) phylogenetic analyses on each of the subsampled alignments using RAxML-VI-HPC version 7.0.4 with the GTRCAT nucleotide substitution model (Stamatakis, 2006). We also performed 200 non-parametric bootstrap replicates (Felsenstein, 1985) for each alignment using the same methods. For the ML search on the full dataset (42,626 sequences), we used a parallelized version of RAxML for IBM BlueGene L clusters (Ott et al., 2007). This analysis took approximately 9 days to run on 256 processors at Iowa State University. A full bootstrap analysis was not feasible using this approach. Therefore, we created 100 nonparametric bootstrap datasets using HyPhy (Pond, Frost & Muse, 2005) and performed a ML analysis on these datasets using FastTree 2.1 with the GTRCAT model (Price, Dehal & Arkin, 2010). The FastTree analyses used four minimum-evolution SPR rounds and the “-mlacc 2—slownni” option to increase the search space of the NNI swaps in the ML analysis. Optimal trees from these analyses are available in the Dryad data repository (Data will be submitted upon acceptance).

### Number of endosymbiont lineages

For all ML and ML bootstrap trees, we inferred the number of independent endosymbiont clades with PAUP* (Swofford, 2003). Endosymbiont genomes degrade over time due to Muller’s Ratchet (Moran, 1996; Moran & Baumann, 2000). This genomic degradation also occurs in louse endosymbionts (Kirkness et al., 2010; Allen et al., 2009). Due to this process, it is unlikely that endosymbionts would be able to revert to a free-living stage. Therefore, we assumed that endosymbiosis is a non-reversible binary character (i.e., non-endosymbionts can become endosymbionts, but endosymbionts cannot become non-endosymbionts) in this analysis. The placement of the root can affect the number of inferred origins of louse endosymbiosis, and the root of all sampled *Gammaproteobacteria* sequences is uncertain. Therefore, we calculated the number of louse endosymbiont origins using every possible rooting of the 16S rDNA tree. Re-rooting was done with a C+ + program written for this analysis. Our estimate of the number of endosymbiont lineages is based on a root that implied the fewest louse endosymbiont origins.

The estimate of louse endosymbiont origins may change with increased taxonomic sampling due to either the insertion of new, non-endosymbiont sequences within an endosymbiont clade or changes in inferred relationships among endosymbionts. To help distinguish between these two possibilities, we took all optimal and bootstrap trees from the 80%, 85%, 90%, 95%, and full datasets and pruned them so that they would have the same taxon sampling as the smaller subsets. For example, the trees from 90% dataset were pruned to create three datasets in which they would have only the taxa from (1) the 85% dataset, (2) the 80%, and (3) the 70% datasets. Then we calculated the number of louse endosymbiont origins for each of the pruned trees. If the number of louse endosymbiont origins in the pruned trees equaled the number of endosymbiont origins estimated from the original datasets with the same taxa, then changes in the number of estimated endosymbiont origins in larger trees are caused by additional taxa breaking up endosymbiont clades. The taxon pruning was done with a Perl script and Newick utilities (Junier & Zdobnov, 2010).

### Simulations

We used a parametric bootstrapping (i.e., simulation) experiment to evaluate if bias or error in our phylogenetic analyses could lead to erroneous estimates in the numbers of louse endosymbiont origins. Specifically, the parametric bootstrapping experiment examined if our phylogenetic analyses could result in estimates of multiple endosymbiont origins if there was actually only a single origin of endosymbiosis. First, for the 70%, 80%, 85%, 90%, and 95% datasets, we performed a ML analysis in RAxML (Stamatakis, 2006) in which all the louse endosymbiont sequences were constrained to a single clade, which would imply a single origin of endosymbiosis. We then estimated the optimal branch length and GTR + I + G substitution model parameters for the 16S rDNA alignment used to infer the ML constraint tree using the resulting ML constraint topology for each dataset and simulated 100 alignments of the same dimensions using HyPhy (Pond, Frost & Muse, 2005). We performed a ML analysis with RAxML and estimated the number of louse endosymbionts on each simulated dataset using the same protocol as we used on the empirical data. We then compared the number of endosymbiont origins inferred from our single-origin simulations to the number of origins inferred from the empirical data.

## Results

### Endosymbiont sequences

We identified 18 endosymbiont sequences from 17 of the 23 louse specimens; two were found in a single louse (*Ancistroplax crocidurae*) and none were found in six specimens (Table 1). We used BLAST searches and AT content to assess if the newly acquired louse bacteria were from an endosymbiont (genomes of endosymbionts are often, but not always, AT-rich; Bentley & Parkhill, 2004; McCutcheon & Moran, 2012). All 18 sequences were most similar to other endosymbiont sequences in BLAST searches, and seven of these were most similar to other confirmed Anoplura endosymbionts (Table 1). All 18 sequences had ≥45% AT content, and 14 had ≥50% AT content, which is consistent with many endosymbionts (Moran & Baumann, 2000). We did not find any *Gammaproteobacteria* sequences that met our criterion in the louse genus *Hoplopleura*; however, we found *Alphaproteobacteria* sequences from the common louse pathogen *Bartonella* in four of the five *Hoplopleura* samples. Since our study was focused on *Gammaproteobacteria*, we did not use the *Alphaproteobacteria* sequences in our analyses. The 18 putative *Gammaproteobacteria* endosymbiont 16S rDNA sequences were combined with 12 sucking louse endosymbiont 16S rDNA sequences from GenBank so that all of the alignments used in the phylogenetic analyses contained 30 endosymbiont sequences from lice (Table 1).

### Endosymbiont phylogeny

**Figure 1:**
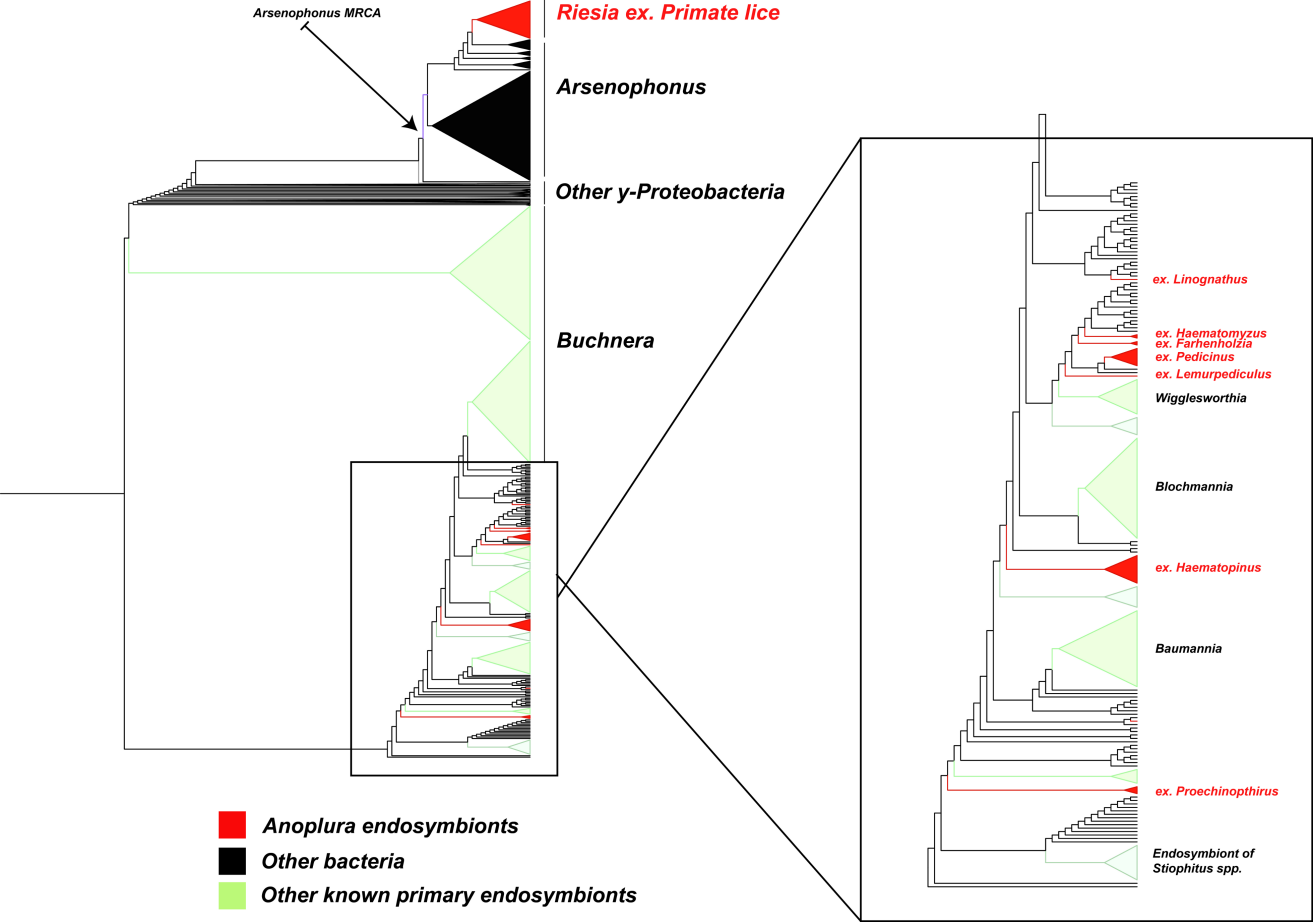
Subset of large phylogenetic tree showing placement and close relatives of endosymbiotic bacteria in Anoplura. A subtree of the full 42,266 *Gammaproteobacteria* tree showing 9 of the 10 endosymbiont lineages from sucking lice (red). For all louse endosymbionts, the louse host genus or group is indicted. All of these sequences cluster together either within or near other known endosymbiont lineages (green) and *Arsenophonus*, a clade of insect bacterial endosymbionts; the arrow points to the Most Recent Common Ancestor (MRCA) of this clade. The 10th lineage of endosymbiont clusters with the genus *Legionella*, which is not shown due to space constraints.

The phylogenetic relationships of the louse endosymbionts were largely consistent with previous studies. Our analysis revealed the same six lineages suggested in earlier publications, with similar topologies for these lineages. For example, endosymbionts from rat lice (*Polyplax* sp.) were nested within the genus *Legionella,* consistent with the findings of Hypsa & Krízek (2007). The endosymbiont lineage *Riesia* was monophyletic with a topology that suggests co-speciation with human, chimp, and gorilla lice (Supplementary Trees), similar to what was found in Allen et al. (2007). The 18 newly sequenced louse endosymbiont lineages revealed new clades of endosymbionts, all of which grouped close to *Arsenophonus* and other known insect endosymbionts including *Baumannia* and *Wigglesworthia* (the endosymbionts of sharpshooters and tsetse flies; Fig. 1).

### Estimates of endosymbiont lineages

**Figure 2:**
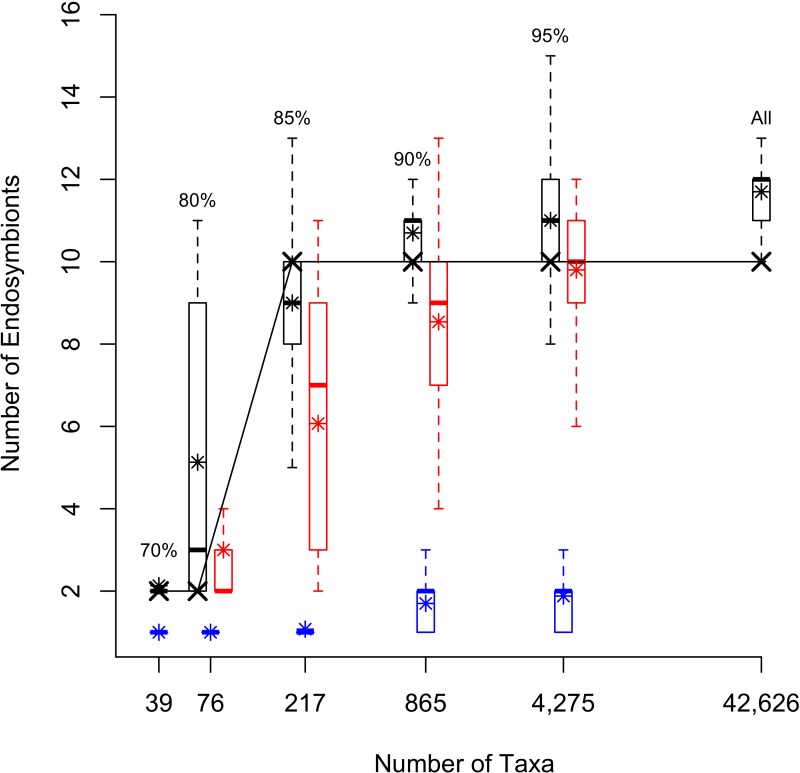
Box Plots showing number of endoysmbiont lineages in differently sampled datasets. The number of sucking louse endosymbiont lineages inferred from phylogenetic trees with different sampling. The number of taxa in each alignment is plotted on a log_10_ scale. Boxplots represent the number of endosymbionts calculated from either the 200 bootstrap replicates for the phylogenetically sampled data sets (in black), across the 100 randomly sampled data sets (red) or the simulated data sets (blue). Boxes represent 50% of the data; whiskers extend to 1.5 times the interquartile range representing 95% of the data, and * shows the average. “X” corresponds to the number of lineages calculated from the ML tree for each data set.

The estimates of endosymbiont lineages increased from 2 in the ML trees from the 70% cluster and 80% cluster datasets with 39 and 76 taxa, respectively, to 10 in the ML trees of the 85%, 90%, 95% clusters, and full datasets with 217, 865, 4,275 and 42,626 taxa, respectively (Fig. 2). For the randomly sampled datasets, the average number of louse endosymbiont lineages increased with the size of the dataset up to 4,275 taxa. For the datasets with fewer than 1,000 sequences, the average estimates from the randomly sampled datasets were smaller than those found from the datasets of equal size that were sampled to maximize sequence diversity (Fig. 2). In the 4,275 taxon randomly sampled dataset, the average number of endosymbiont lineages was similar to the estimate from the phylogenetically sampled dataset with the same number of taxa (9.8 ± 1.3 SD and 10, respectively; Fig. 2).

**Figure 3:**
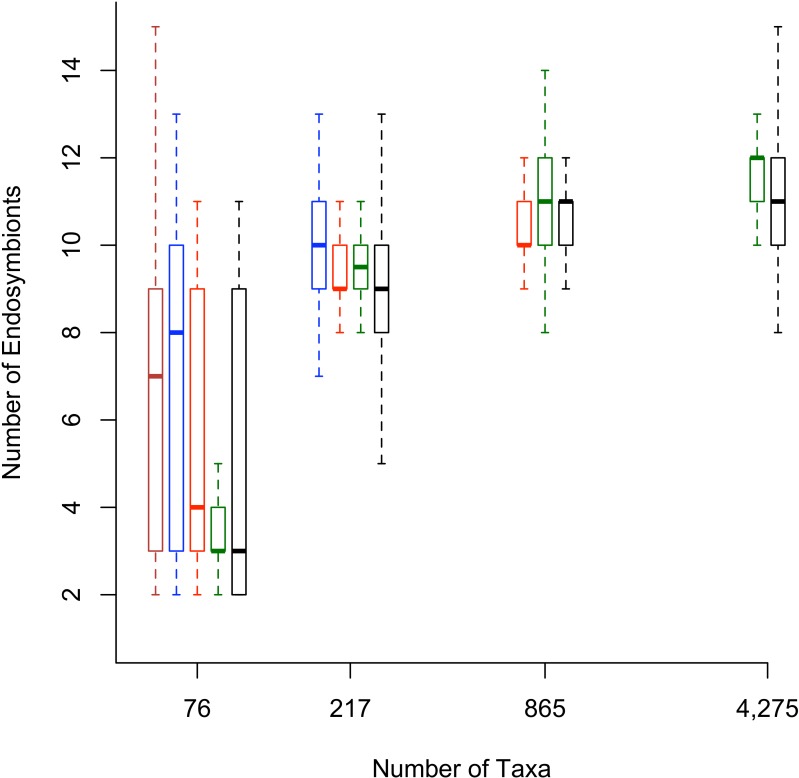
Box plots with number of lineages for reduced phylogenetic trees. The number of sucking louse endosymbiont lineages found for reduced phylogenetic trees. Boxplots represent the number of endosymbiont lineages calculated from 200 bootstrap replicates for the data sets. The 200 bootstrap trees for each data set were then pruned to the taxa found in the smaller data sets and the number of endosymbiont lineages counted. The original data sets are plotted in black. The reduced full data sets are in green, reduced 95% data sets are in red, reduced 90% data sets in blue, and reduced 85% data sets in brown. Boxes represent 50% of the data and whiskers extend to 1.5 times the interquartile range, representing 95% of the data.

Secondly, when the bootstrap replicates from the full dataset were pruned to include only the sequences from the smaller datasets, the number of inferred endosymbiont lineages was similar to the original smaller size datasets (Fig. 3). These results suggest that as more sequences are added to the analyses, the numbers of endosymbiont lineages are changing because the new 16S rDNA sequences break up clades of endosymbionts, not because the new 16S rDNA sequences are changing the backbone topology of the tree.

Finally, we found notable variation in estimates of endosymbiont lineages across the bootstrap replicates (Fig. 2). The standard deviation of number of lineages among bootstrap replicates was lowest for the 70% dataset and highest for the 80% dataset. The parametric bootstrapping analysis of the datasets simulated from a tree with a single origin of endosymbiosis in lice resulted in estimates of the number of endosymbiont lineages ranging from one (for the smaller datasets) to at most three endosymbiont lineages for the larger datasets (Fig. 2). This result indicates that if there was only a single origin of endosymbiosis, we would expect our estimates from 16S rDNA would reflect at most only a few origins. Because the empirical estimates of endosymbiont origins far exceed the estimates in the single-origin simulation, it is likely that there were multiple origins of endosymbiosis in sucking lice.

## Discussion

Phylogenetic trees of bacteria have helped reveal the origins of symbioses and the co-evolutionary history between these organisms and their hosts. While the abundance of 16S rDNA sequences enables us to build enormous phylogenetic trees of bacteria, few studies have explored how sampling of available 16S rDNA sequences affects our interpretations of the co-evolutionary history of bacteria and their hosts. New bacterial sequences can change estimates of endosymbiont lineages, either by adding new endosymbiont lineages, adding non-endosymbionts that break up endosymbiont clades into multiple clades, or changing the topology of the bacterial tree. Therefore, it is important to explore how taxon sampling affects our estimates of endosymbiont lineages.

Overall, our estimates of endosymbiont lineages remain relatively unchanged as long as the tree contains a minimal level of genetic diversity of *Gammaproteobacteria*. For example, once we sampled ∼200 sequences by maximizing sequence diversity, adding additional sequences had little effect on our estimates of the numbers of louse endosymbiont lineages (10; Fig. 2). In contrast, if we added randomly selected sequences, we needed to sample at least ∼4,000 sequences before the estimates of endosymbiont lineages converged to 10 (Fig. 2). This result emphasizes the importance of addressing the question of number of independent endosymbiont origins in the context of all *Gammaproteobacteria* sequence diversity. If the 16S rDNA sequences are chosen to maximize their diversity, fewer sequences may be needed to infer the number of endosymbiont lineages.

16S rDNA is the barcoding gene used to identify unique bacterial lineages, and much of our understanding of bacterial diversity comes from this gene (Klindworth et al., 2013). Therefore, it is uniquely useful for estimating the total number of endosymbiont lineages among *Gammaproteobacteria*. However, phylogenetic trees with thousands of leaves constructed from a single locus likely include much error and uncertainty. It is unclear how much this topological error or uncertainty affects estimates of the number of endosymbiont lineages. We addressed this question using nonparametric and parametric bootstrapping experiments. First, we calculated the number of implied louse endosymbiont origins on all bootstrap trees to assess how topological uncertainty might affect the analyses. Although the estimates of endosymbiont lineages varied among bootstrap replicates (Fig. 2), no bootstrap replicate in the datasets with more than 865 taxa implied fewer than 8 louse endosymbiont lineages. In other replicates, the number of estimated endosymbiont lineages exceeded 15, suggesting that error can inflate estimates of endosymbiont origins (Fig. 2).

We also performed a parametric bootstrapping experiment to assess the number of endosymbiont origins we would infer if there were only a single origin in sucking lice. In some cases, analyses of the simulated datasets inferred more than a single origin, but they never inferred more than three origins on any simulated dataset (Fig. 2). This suggests that error in the topology cannot account for the high estimates of the number of origins of endosymbiosis.

Our work demonstrates that the number of inferred endosymbiont lineages may be accurate if the diversity of sequence sampling is sufficient. Still, it is unclear how many more endosymbiont lineages we would find with greater sampling, and unlike many multicellular eukaryote lineages, the amount of existing bacterial diversity is unclear. Adding any single new sequence from other *Gammaproteobacteria* could reveal additional endosymbiont origins. Notably, our sampling represents only a small fraction (5%) of the total number of anopluran species currently recognized. Additional sampling may reveal more endosymbiont lineages, and it will only be possible to estimate the true number of endosymbiont lineages in this group with greater sampling. Additionally, it is possible that individual louse lineages have acquired multiple endosymbiont lineages, especially considering that endosymbiont genomes are known to degrade over time ( Moran, 1996; Moran & Baumann, 2000; Kirkness et al., 2010; Allen et al., 2009). Therefore, we may be underestimating the number of sucking louse endosymbiont lineages. Regardless, our estimate of at least 10 endosymbiont origins is large compared to other insect/endosymbiont assemblages with one or only a few endosymbiont lineages (e.g., aphids and *Buchnera*; Moran & Baumann, 1994).

Although it is impossible to determine with certainty the nature of the relationship of the bacteria with the host (e.g., mutualistic primary endosymbionts or facultative) from only 16S rDNA sequences, our results suggest the possibility that co-evolution of bacterial endosymbionts in sucking lice is an extremely labile process. Further, with more extensive sampling it may be possible to determine the sister taxa of all of the symbiont lineages and start to form hypotheses about the origin of the endosymbionts. In this analysis we found that the sister taxon to each endosymbiont was different in most trees, therefore it was not possible to accurately determine the sister species for each endosymbiont. In the future, building more robust trees with high support values on all nodes (possibly using more genes or genome sequences) may shed some light onto the origins of these endosymbionts.

While 16S rDNA is, and will likely be for the foreseeable future, the most widely sequenced gene for bacterial identification, additional genes and even genomic sequencing will enable phylogenetic estimates of the bacteria based on many loci. Although these data may ameliorate biases or error associated with 16S rDNA and reduce uncertainty in phylogenetic estimates, they will unlikely rival the diversity found in 16S rDNA, which may be critical for estimating the number of endosymbiont lineages. In the future, combining the sampling of 16S rDNA with the phylogenetic power of large genomic data will likely provide a more complete picture of the evolutionary history of insect associated bacteria.

##  Supplemental Information

10.7717/peerj.2187/supp-1 Supplemental Information 1 Alignment, trees and sequences

## Additional Information and Declarations

### DNA Deposition

The following information was supplied regarding the deposition of DNA sequences: GenBank: KX146199–KX146216.

### Data Availability

The following information was supplied regarding data availability: Dryad DOI: 10.5061/dryad.db0r1.
